# Ileo-Ileal Knot with Three Closed Loops: A Case Report and Review of Pathophysiology

**DOI:** 10.70352/scrj.cr.25-0011

**Published:** 2025-05-08

**Authors:** Ryogo Ito, Hideo Matsubara, Masahito Uji, Yasutomo Miura, Yudai Aoki, Ryoichi Shimizu, Mayuko Fujisawa, Yasuji Mokuno

**Affiliations:** Department of Surgery, Yachiyo Hospital, Anjo, Aichi, Japan

**Keywords:** ileo-ileal knot, intestinal knot, intestinal volvulus, strangulated bowel obstruction, closed loop

## Abstract

**INTRODUCTION:**

The ileo-ileal knot is a rare condition in which 2 ileal loops form a knot, leading to obstruction, ischemia, and the need for urgent surgical intervention. It is challenging to diagnose preoperatively, with 2 closed loops on CT scan as its characteristic feature. This case revealed 3 nearly situated closed loops, which offers insights into the diagnosis and management of the ileo-ileal knot.

**CASE PRESENTATION:**

A 69-year-old female patient presented with a 3-h history of upper abdominal pain and repeated vomiting. Contrast-enhanced CT scan revealed a poorly enhanced small-bowel obstruction, with 3 nearly situated closed loops. The patient was diagnosed with a strangulated small-bowel obstruction, likely caused by an unusual internal hernia. Emergency laparoscopic surgery was performed. Due to the difficulty in assessing the intestinal condition and the risk of injury, the procedure was converted to open surgery. Direct evaluation during laparotomy confirmed the diagnosis of an ileo-ileal knot. The knot was untied, further improving bowel color and peristalsis and allowing bowel preservation. Based on the retrospective evaluation of the reconstructed CT scan images and intraoperative findings, the 3 closed loops comprised 2 tied loops and 1 knotting loop. The patient initially recovered. However, she was readmitted on postoperative day 24 due to bowel obstruction caused by fibrosis near the terminal ileum. After undergoing ileocolic resection, the patient recovered uneventfully.

**CONCLUSIONS:**

An ileo-ileal knot should be considered in cases of strangulated bowel obstruction with more than 2 closed loops. Because the intraoperative assessment of the knot is challenging, laparoscopic surgery can be difficult. Therefore, early conversion to laparotomy should be considered.

## INTRODUCTION

Intestinal knotting is a rare condition characterized by the knotting of 2 intestinal loops, resulting in obstruction and ischemia. It is most commonly observed between the ileum and the sigmoid colon. Nevertheless, an ileo-ileal knot involving the ileum alone is rare.^1–3)^ Early surgical intervention is typically required because delayed diagnosis can result in severe complications or mortality.

The condition is challenging to diagnose preoperatively, and CT scan is an important diagnostic tool. Previous studies have shown the presence of 2 closed loops on CT scan, which is a characteristic feature of intestinal knotting.^4,5)^ However, in the current case, there were 3 closed loops associated with an ileo-ileal knot, a finding that deviates from the patterns previously described.

This report explored the mechanisms underlying the formation of 3 closed loops and their relevance to diagnostic and therapeutic approaches.

## CASE PRESENTATION

A 69-year-old female patient presented to the emergency department due to a 3-h history of upper abdominal pain and repeated vomiting. She had no previous history of abdominal surgery. Upon admission, her vital signs were stable: blood pressure, 186/91 mmHg; heart rate, 66 bpm; oxygen saturation on room air, 100%; and body temperature, 36.3°C. Physical examination revealed a flat and soft abdomen with rebound tenderness in the periumbilical region. The laboratory findings showed a normal white blood cell count and C-reactive protein levels (**Table 1**).

**Table 1:** Laboratory findings on admission

Parameter		Value
Hematology	White blood cell count, ×10^3^/μL	5.8
	Hemoglobin, g/dL	14.3
	Hematocrit, %	40.8
	Platelet count, ×10^3^/μL	236
Biochemistry	C-reactive protein, mg/dL	0.01
	Blood urea nitrogen, mg/dL	19
	Creatinine, mg/dL	0.75
	Total protein, g/dL	7.3
	Albumin, g/dL	4.5
	Aspartate aminotransferase, U/L	28
	Alanine aminotransferase, U/L	22
	Lactate dehydrogenase, U/L	229
	Total bilirubin, mg/dL	0.7
Electrolytes	Sodium, mmol/L	138
	Potassium, mmol/L	3.9
	Chloride, mmol/L	99

The patient’s laboratory findings on admission are shown in the table. White blood cell count and C-reactive protein levels were within the normal range.

**Fig. 1:**
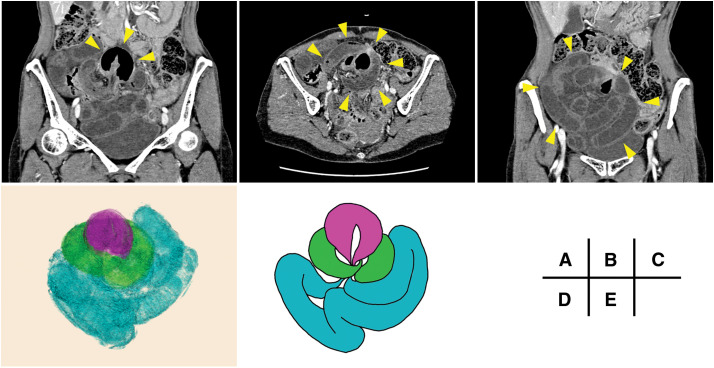
Contrast-enhanced CT scan images and reconstructed 3D visualization of the ileo-ileal knot. (**A**) Coronal CT scan image showing the first small, closed loop (arrowhead). (**B**) Axial CT scan image revealing the second closed loop (arrowhead). (**C**) Coronal CT scan image indicating the third closed loop (arrowhead). (**D**) 3D reconstruction of the strangulated bowel, with the first closed loop shown in red, the second in green, and the third in blue. (**E**) Schematic image of the 3D reconstruction shown in (**D**).

Contrast-enhanced CT scan revealed small-bowel obstruction with poor enhancement, which formed three closely situated closed loops. The first closed loop was observed on coronal CT scan image (**Fig. 1A**), the second on axial CT scan image (**Fig. 1B**), and the third on another coronal CT scan image (**Fig. 1C**). These loops were indicative of a complex strangulated bowel obstruction, which was initially interpreted as an unusual internal hernia. Laparoscopic surgery was initiated 1 h after the CT scan.

**Fig. 2:**
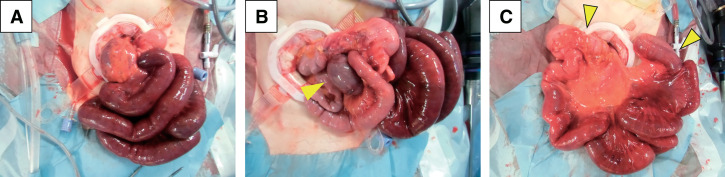
Intraoperative findings of the ileo-ileal knot. (**A**) Ischemic small-bowel with a length of approximately 1 m, located 5 cm proximal to the terminal ileum. (**B**) Incarcerated bowel segment with a length of about 10 cm that was observed dorsally, highlighted by arrowheads. (**C**) Complete release of all strangulations after cautious manual reduction of the dorsal bowel. The bowel segment resected during the second surgery corresponded to the area marked between the arrowheads.

During laparoscopic surgery, we attempted to relieve the strangulation. However, due to the risk of bowel injury and difficulty in fully assessing the intestinal condition, the procedure was converted to open surgery. Small-bowel ischemia, with the affected area measuring approximately 1 m, located 5 cm proximal to the terminal ileum, was identified (**Fig. 2A**). In addition, the incarcerated bowel that measured about 10 cm was found dorsally (**Fig. 2B**). All strangulations were successfully released with cautious manual reduction of the dorsal bowel (**Fig. 2C**). Internal hernia was not observed, and the intraoperative diagnosis was an ileo-ileal knot. As the bowel color improved after release of the strangulations, small-bowel resection was not performed.

The patient recovered uneventfully, and she was discharged on postoperative day 8. However, on postoperative day 24, she was readmitted due to a bowel obstruction near the terminal ileum. Conservative management was unsuccessful, and a second surgery was performed on postoperative day 42. Intraoperatively, severe adhesions involving the terminal ileum and cecum, which led to the formation of a single mass, were identified. Because preservation was deemed unfeasible, an ileocolic resection was performed. The resected bowel segment, corresponding to the area marked by arrows in **Fig. 2C**, encompassed the entire portion of the small bowel involved in the knot during the initial surgery. Pathological examination revealed fibrosis caused by ischemic changes. The patient recovered well after the second surgery, and she was discharged on postoperative day 8 without further complications.

## DISCUSSION

An intestinal knot is a rare and serious condition in which 2 segments of the intestine become tied together, resulting in intestinal obstruction and impaired blood flow. This anomaly can occur in different parts of the intestine, with the most common type being the ileo-sigmoid knot, where loops of the ileum wrap around the bottom of a redundant sigmoid loop. The ileo-ileal knot is a rare condition where 2 segments of the ileum form a knot.^1–3)^

The exact cause of ileo-ileal knotting is not completely understood. However, several anatomical and dietary factors have been proposed. The anatomical factors include decreased mesenteric fat, an oversized mesentery, and partial bowel fixation caused by adhesions, which may facilitate abnormal bowel mobility and looping.^5–7)^ In terms of dietary factors, a high-fiber diet, rapid food intake, and fasting may contribute to the development of the condition by altering gastrointestinal motility.^5,8)^ Further studies are required to elucidate the role of these mechanisms.

An ileo-ileal knot is challenging to diagnose preoperatively. In most cases, the condition is diagnosed intraoperatively. A literature search using PubMed and Google Scholar yielded no reported cases of an ileo-ileal knot diagnosed preoperatively. However, CT scan is a valuable tool for preoperative diagnosis, with the presence of a double closed loop being a characteristic finding.^4,5)^ This represents 2 intestinal loops tied at a single focal point, forming closed loops.

In our case, 3 closed loops were identified on preoperative CT scan. However, it was diagnosed as an unusual internal hernia. An ileo-ileal knot was not recognized or considered as a differential diagnosis. Based on the retrospective analysis of the CT scan findings and the associated pathophysiology, the following mechanism was proposed: **Fig. 1D** shows a 3D reconstruction of the bowel loops, and **Fig. 1E** depicts the corresponding schema. The strangulated bowel segment (green) formed a ring, with 2 additional closed loops (red and blue) on either side. Intraoperatively, all the strangulations were resolved successfully with the release of a small, strangulated bowel segment (red). Based on these findings, the 3 loops comprised 2 tied loops and 1 knotting loop, which formed the knot itself.

**Fig. 3:**
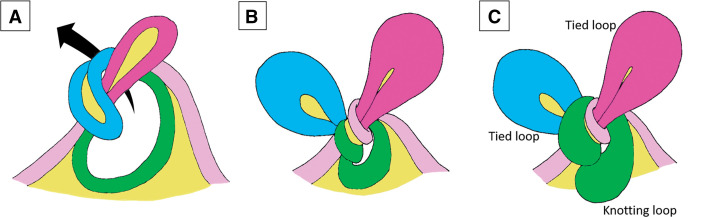
Conceptual diagram of the pathophysiology of an ileo-ileal knot forming 3 closed loops. (**A**) Initial wrapping of 1 bowel segment (blue) around another (red), which caused the formation of the knot. (**B**) Development of 2 closed loops (tied loops, highlighted in blue and red) as the knot tightens. (**C**) Expansion of the knot itself (knotting loop, highlighted in green), which created a third closed loop.

**Fig. 3** illustrates a conceptual mechanism derived from the observed CT findings and intraoperative course. The colors used for the bowel segments in this figure are intentionally matched to those in **Fig. 1**, representing the 2 tied loops and the knotting loop. Initially, one segment of the bowel (blue) wraps around another (red), as shown in **Fig. 3A**. This resulted in the formation of an intestinal knot, creating 2 closed (tied) loops, as depicted in **Fig. 3B**. Subsequently, the knot itself expanded, forming a third closed loop (green), referred to as the knotting loop (**Fig. 3C**). Based on the proposed pathophysiology, an ileo-ileal knot should be considered in the differential diagnosis of strangulated bowel obstruction when more than 2 closed loops are observed on CT scan.

There are no reported cases of an ileo-ileal knot successfully treated with laparoscopy. In the case reported by Taniguchi et al.,^9)^ laparoscopy was used for diagnostic purposes only before promptly converting to open surgery. Recent studies have reported the application of laparoscopic surgery for strangulated bowel obstruction.^10,11)^ In our case, the surgery was initiated laparoscopically. However, due to difficulties in understanding the pathophysiology and the risk of bowel injury, the procedure was converted to open surgery. The pathophysiology was assessed accurately via direct visualization during open surgery, and the knot was untied successfully.

However, the decision to preserve the bowel should be further considered. In the study of Umetsu et al., 11 of 13 patients required bowel resection.^6)^ In our case, improvement in bowel color and peristalsis was observed after releasing the strangulation. Thus, bowel preservation was considered feasible. Necrosis did not occur because the knot was relatively loose. However, bowel fibrosis necessitated subsequent resection. Although bowel preservation is ideal, ileo-ileal knotting often leads to high probability of requiring bowel resection.

Considerably, we propose the following treatment strategy: when strangulated small-bowel obstruction is detected on CT, careful assessment of the number of involved loops is essential. If 2 or more loops are intertwined, ileo-ileal knotting should be considered in the differential diagnosis. In hemodynamically stable patients, laparoscopic surgery can be initiated. However, laparoscopic release of the knot presents several challenges: (1) identifying the appropriate bowel loop to manipulate successful untangling, (2) minimizing the risk of bowel injury due to excessive force during instrument handling, and (3) preserving ischemic bowel segments whenever feasible. Given these complexities, if laparoscopic manipulation proves difficult, early conversion to laparotomy should be strongly considered to ensure a safe and effective surgical outcome.

## CONCLUSIONS

We report a case of an ileo-ileal knot with 3 closed loops. When a strangulated bowel obstruction with more than 2 closed loops is observed, an ileo-ileal knot should be considered as the differential diagnosis. Laparoscopic surgery can be an option; however, due to the challenges associated with knot release, early conversion to laparotomy should be promptly undertaken when required.

## DECLARATIONS

### Funding

None.

### Authors’ contributions

All authors contributed to the treatment and management of the patient.

The second author provided supervision and guidance throughout the study.

All authors were involved in drafting the manuscript, critically revising it for important intellectual content, and approving the final version for submission.

### Availability of data and materials

The dataset supporting the conclusions of this article is included within the article.

### Ethics approval and consent to participate

This study was conducted in accordance with the principles of the Declaration of Helsinki. The Institutional Review Board for Clinical Research of Yachiyo Hospital waived the need for ethical approval.

### Consent for publication

A written informed consent for the publication of this case report was obtained from the patient.

### Competing interests

The authors declare that they have no competing interests.
